# The Malaria System MicroApp: A New, Mobile Device-Based Tool for Malaria Diagnosis

**DOI:** 10.2196/resprot.6758

**Published:** 2017-04-25

**Authors:** Allisson Dantas Oliveira, Clara Prats, Mateu Espasa, Francesc Zarzuela Serrat, Cristina Montañola Sales, Aroa Silgado, Daniel Lopez Codina, Mercia Eliane Arruda, Jordi Gomez i Prat, Jones Albuquerque

**Affiliations:** ^1^ Federal Rural University of Pernambuco Department of Statistics and Informatics Recife Brazil; ^2^ Federal University of Rio Grande do Norte Department of Informatics and Applied Mathematics Natal Brazil; ^3^ Universitat Politècnica de Catalunya BarcelonaTech Barcelona Spain; ^4^ Vall dHebron University Hospital Microbiology Department Barcelona Spain; ^5^ Aggeu Magalhães Research Center FIOCRUZ Recife Brazil; ^6^ Keizo Asami Laboratory of Imunopathology Federal University of Pernambuco Recife Brazil

**Keywords:** artificial intelligence, applied computing, automated diagnosis, malaria, mobile devices

## Abstract

**Background:**

Malaria is a public health problem that affects remote areas worldwide. Climate change has contributed to the problem by allowing for the survival of Anopheles in previously uninhabited areas. As such, several groups have made developing news systems for the automated diagnosis of malaria a priority.

**Objective:**

The objective of this study was to develop a new, automated, mobile device-based diagnostic system for malaria. The system uses Giemsa-stained peripheral blood samples combined with light microscopy to identify the Plasmodium falciparum species in the ring stage of development.

**Methods:**

The system uses image processing and artificial intelligence techniques as well as a known face detection algorithm to identify Plasmodium parasites. The algorithm is based on integral image and haar-like features concepts, and makes use of weak classifiers with adaptive boosting learning. The search scope of the learning algorithm is reduced in the preprocessing step by removing the background around blood cells.

**Results:**

As a proof of concept experiment, the tool was used on 555 malaria-positive and 777 malaria-negative previously-made slides. The accuracy of the system was, on average, 91%, meaning that for every 100 parasite-infected samples, 91 were identified correctly.

**Conclusions:**

Accessibility barriers of low-resource countries can be addressed with low-cost diagnostic tools. Our system, developed for mobile devices (mobile phones and tablets), addresses this by enabling access to health centers in remote communities, and importantly, not depending on extensive malaria expertise or expensive diagnostic detection equipment.

## Introduction

Malaria is a public health problem worldwide. A total of 214 million cases of malaria occurred globally in 2015 with 438,000 deaths [1]. In 2014, 97 countries reported the continuous transmission of malaria and the World Health Organization (WHO) estimates that around 3.2 billion people are at risk of becoming infected with this disease [1]. In most of the high-burden countries, weak healthcare systems contribute to slower than average declines in malaria incidences and high rates of mortality [1]. Therefore, early and accurate diagnosis of malaria is essential for effective disease management and surveillance. Indeed, misdiagnosis can result in significant morbidity and mortality. In addition, the correct diagnosis of patients with febrile illnesses may help to reduce the emergence and spread of drug resistance by reserving antimalarial treatments for those with a malaria diagnosis.

Malaria is currently diagnosed by microscopy with stained slides (thick blood films or blood smear) or by rapid diagnostic tests (RDTs) [2,3]. The thick blood film method of slide staining is the most common and inexpensive technique to diagnose malaria, whereas, the blood smear method is specifically used to identify the morphology and species of the *Plasmodium* parasite [4]. Today, it is essential that malaria diagnosis technicians are experienced in identifying species of *Plasmodium* using these techniques. For this reason, RDT methods are quite effective and widely used in some regions (ie, the Brazilian Amazon). However, RDT methods are expensive and not always effective in identifying samples with mixed species [3,5]. Furthermore, scientists are concerned about parasite resistance to antimalarial medicines and mosquito vector anopheles to insecticides [1]. Thus, a fast, in-place diagnosis system is essential to control malaria.

In recent decades, a number of researchers, including those from computing areas, have sought cost-effective solutions to assist health professionals in the control of epidemics and diseases. For example, Leal Neto et al (2014) developed a real-time diagnostic system for epidemiological events simulations [6]. Medical imaging has also been used successfully in the diagnosis of diseases. Kaewkamnerd and colleagues (2012) developed a 5-phase image analysis system to detect and classify malaria [7]. Techniques, such as hue-saturation-value (HSV) and adaptive threshold, have been used to extract image characteristics and automated systems of image capturing using a motor adapted to a microscope have been proposed [8]. In another study, Anggraini et al (2011) developed an application to successfully separate background of blood cells by solving image segmentation problems [9].

Here, we propose a low-cost, automated diagnostic system for malaria. Digital processing image techniques and a learning process based on artificial intelligence algorithms were combined to develop the system. Prior to app development, training and validation of the classifier were implemented on a personal computer in C++ language with Microsoft Windows 8.1. The minimum requirements for the app were that is used an Android operating system of 4.2 or higher and had a rear camera of at least 5 megapixels (MPs). Therefore, the Galaxy Tab 2 was used for testing. Taking advantage of this computing infrastructure, the system aims to aid public health officials in remote locations by trying to solve pending issues such as accessibility, cost, rapidness, and accuracy in malaria diagnosis.

## Methods

The facial recognition method proposed by Viola and Jones is known as a heuristic method for the robust, fast, and accurate detection of faces in images [10]. Indeed, several studies have demonstrated the application of the technique [11-13]. The Viola and Jones' facial recognition method was used to develop a new method for the representation of images called integral image, which makes use of a simple and efficient classifier using an adaptive boosting learning algorithm [14]. It has also been used in the development of the cascade of classifiers method. This method uses a constant computational cost , which enables its use in real-time applications. The steps of the algorithm will be presented in the following sections.

### Haar-Like Features

**Figure 1:**
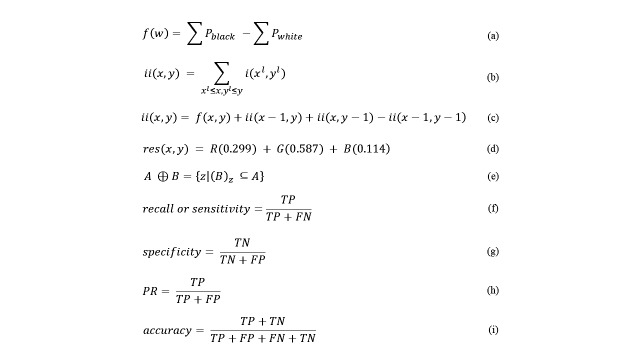
Equations.

Historically, direct pixel manipulation has been a computationally complex problem [15]. As such, Viola and Jones [10] aimed to build a system that could be executed in constant time. They suggested an adaptation of the basic functions of haar described by Papageorgiou and colleagues [16], and began to use haar-like features added to the use of the integral image. Haar-like features are rectangles of white and dark regions. The features value is given by the difference between the sum of the intensities of the pixels of the light region (white) and the sum of pixel intensities in dark region (black) (equation a, Figure 1) where *f(w)* is a value of feature in the windows *w*, Σ *P*_black_ is the sum of pixels in the black region, and Σ *P*_white_ is the sum of pixels in the white region.

**Figure 2:**
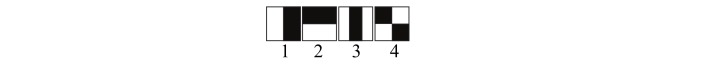
Haar-like features.

Four basic haar-like features are described to face-detection problems (Figure 2). Each resource-type of haar-like features indicates attendance or absence of features in the image, such as edge detection, texture, and others. The standard detector is used in 24 x 24 pixels, generating up to 160,000 rectangle features by subwindow [10,16].

### Integral Image

Integral image is an intermediate representation to quickly calculate the sum of values in a rectangular subset of a grid. The algorithm is used to calculate pixel function in real numbers *f(x,y)* (ie, to calculate pixel intensity along a rectangular region of the image). For a fast calculation of haar features’ rectangles, Viola and Jones [10] proposed the use of integral image (equation b, Figure 1), where *ii(x,y)* is the integral image and *i(x,y)* is the original image. For each point *(x,y)* the value of the sum of all pixels higher and to the left is assigned, such that *x*^1^≤ *x,y*^1^≤ *y.*

The integral image can be obtained with a single pass over the image, using sum area value added in *(x,y)* (equation c, Figure 1). Once an integral image is computed, the evaluation of any area of the rectangle can be performed in constant time, using only four image references.

### Adaptive Boost Algorithm

Viola and Jones [10] used the idea of weak classifiers, *h(x,f,p,θ)*. In that definition, there is a simple structure containing a subwindow *(x)* that consists of a feature *(f)*, threshold *(θ)*, and parity *(p)*.

The learning algorithm selects a unique feature that best separates the positive and negative examples. This feature was based on a machine learning meta-algorithm proposed by Freund and Schapire [14]. The outputs of the learning algorithm are called weak classifiers. These weak classifiers were combined with a weighted sum that represented the final result of the potential classifier. Its main feature was the distribution of weights in sets of examples and the distribution change over the course of the algorithm iterations [10].

### Cascade of Classifiers

In the learning tasks, a high cost was given for evaluating the entire training set. While the reduction in the number of classifiers tended to improve the speed, the decrease of classifiers reduced the hit hat [15]. Viola and Jones proposed a solution to get a choice of good classifiers in a large set by creating decision trees called cascades of classifiers [10]. In the initial stage, the cascade of classifiers searched the simplest classifiers (generic) and rejected the greatest number of potential negative subwindows. The algorithm began with few features and, as it advanced, the number of cascade stages increased. With more stages the number of classifiers grew and the accuracy improved. In the detection of faces, the best cases are given between 15 to 25 stages [10].

### Image Processing

Image processing techniques seek to improve the appearance or simplify an image, by correcting and/or eliminating noise that arose during image acquisition (equipment) or as a result of image degradations (lighting problems). Domains that are given to highlight on images are space, which refers to the plane of the image working directly on top of the pixels and frequency, which are changes in the images after Fourier transformation [17]. The interpretation of digital data is a computationally complex task with a high computational cost [8]. The segmentation or partitioning of digital images into smaller parts is thus an essential task to assist the image interpretation process and images, in general, often contain distinct features that can complicate this process.

Differently-sized images, distorted shapes, lighting problems, and other characteristics contribute to image noise. Image noise, along with certain data contained in images can hamper the identification of features. For example, the image capture of car license plates occurs in uncontrolled environments (ie, lighting variations by virtue of time and weather conditions) [17]. The simplest and most widely used image segmentation technique is binarization which consists of classifying the pixels of a given image according to one or several thresholds. This technique is widely used to separate the background from the objects of interest. In global binarization, cutting a single threshold value is set for the entire image. This is advantageous because it improves processing time; however, image quality may be poor because of potential image noise. As such, global binarization is more suitable for controlled environments. On the other hand, local binarization allows for different cutoff points to different regions of the image. A disadvantage of this technique is the high processing time [14,18].

### Experimental Approach

**Figure 3:**
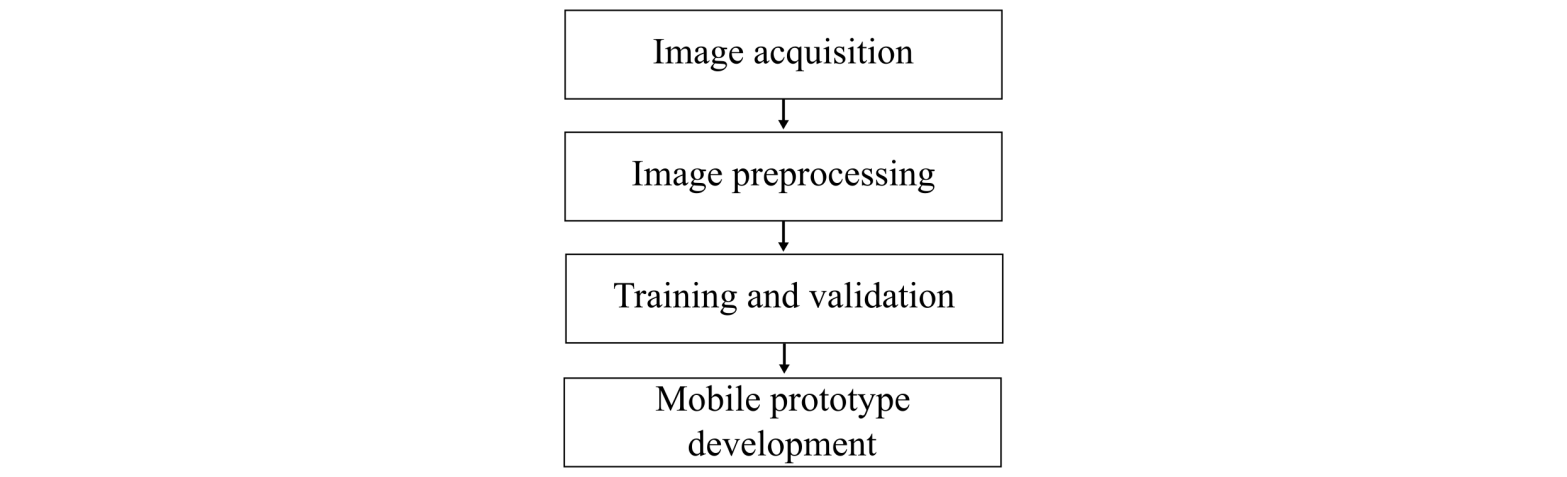
Flowchart of the experimental model.

We developed a mobile device-based automated system to detect malaria. The algorithm specifically identifies *Plasmodium falciparum* in the trophozoite ring stage. Preprocessing steps were used to improve the quality of the microscopy images (eg, Otsu [19]), and Gaussian filters [20] and mathematical morphology techniques [14] were used for training and classification. With respect to image acquisition, preprocessing, training, and validation were performed on a personal computer (Intel Core i3-3217U 1.8 GHz 4096MB RAM) prior to the development of the mobile app (Android 4.2 or higher). The flow of activities in the experimental model is illustrated in Figure 3.

### Image Acquisition

An image acquisition step was necessary in the development of the experimental model. The Microbiology Department (Drassanes Unit) of Vall Hebron Hospital, Barcelona, Spain and the microbiology lab of the Research Center Aggeu Magalhães(FIOCRUZ) Recife, Brazil have a collection of about 500 slides from patients diagnosed with malaria. All the images obtained were classified by experimental parasitologists. This collection was used to perform the image acquisition for the diagnosis algorithm.

**Figure 4:**
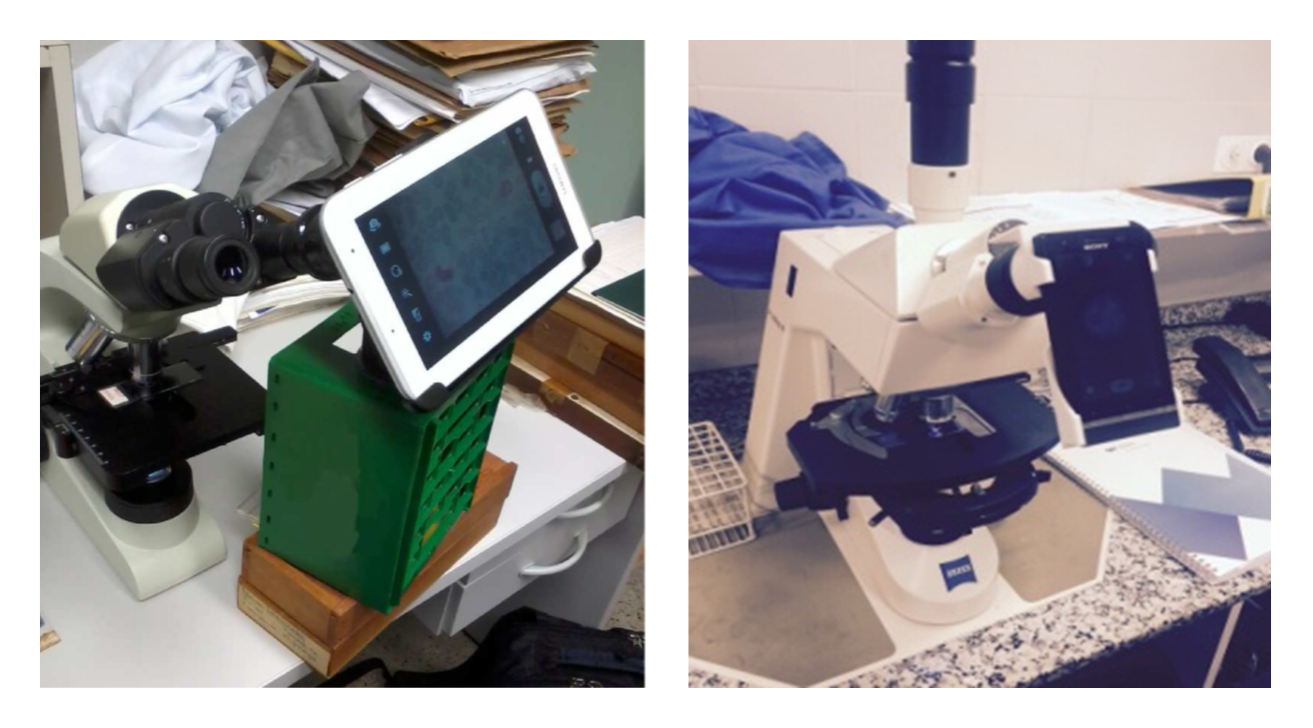
Polyvinyl chloride (PVC) was used as support for the tablet (left) and mobile phone (right).

Malaria parasites require magnification of at least 1000 times for their identification in blood smears [1]. A light microscopy was used along with the Logitech c270 (webcam), the Samsung Galaxy Tab 2 (mobile phone), and the Sony DSC H1 (semi-professional camera). Polyvinyl chloride (PVC), low-cost support pieces (less than US $1) were designed to attach the devices to the light microscopes and to the 3D printer. The image acquisition stage is illustrated in Figure 4.

### Image Preprocessing

**Figure 5:**
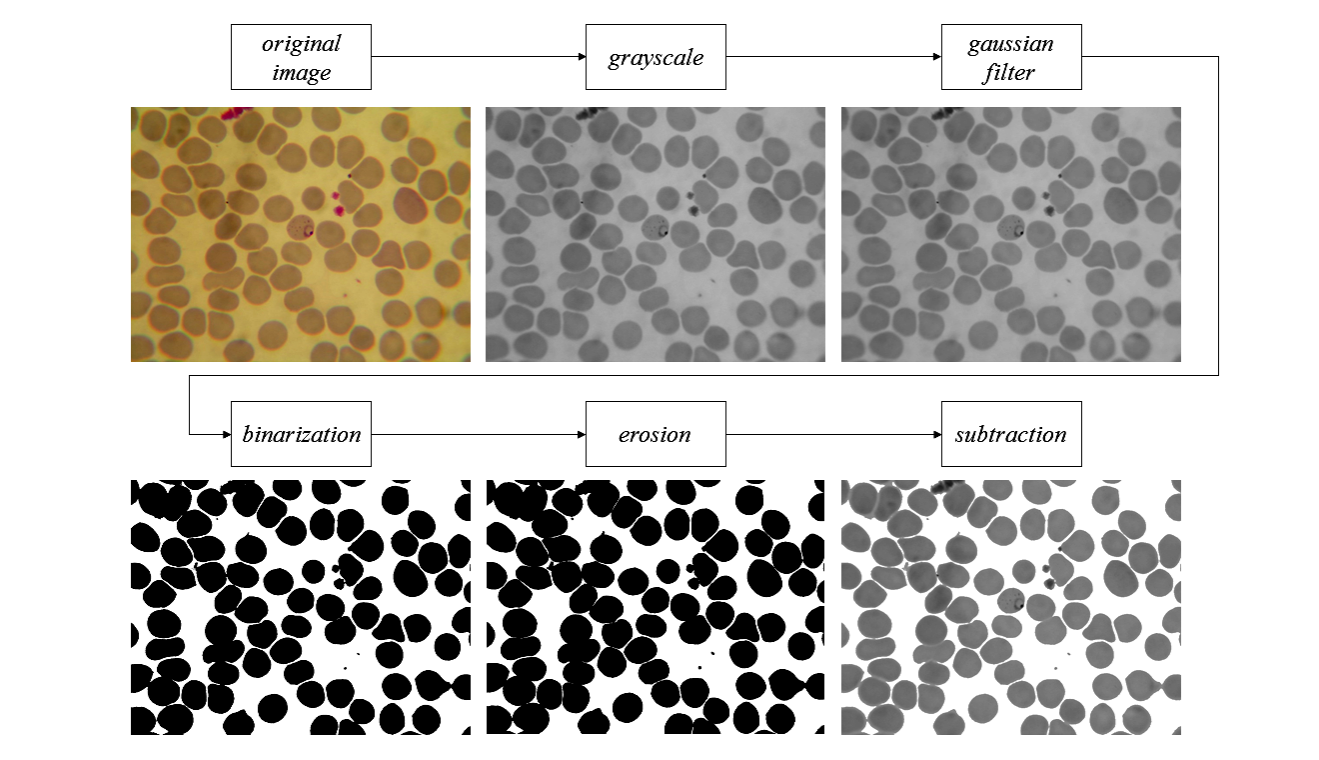
Image preprocessing steps.

In the preprocessing step, some improvements in the images were made to limit the scope or search space for the classification algorithm. The preprocessing step is shown in Figure 5. The original image was given in the red, green, blue (RGB) color model. This model was used because it is the most popular and simple to implement. In addition, since RGB is used in devices such as monitors, video cameras, and mobile phones, conversions to other color models was not required [8]. For this step, the original image was acquired through the blood smear method [1].

The objective of this preprocessing step was to reduce the search scope, such as separate blood smears of the background. In this study, we did not use the entire RGB color space model, since performing segmentation operations on images with several layers of colors can be quite expensive computationally [8]. The original image was thus converted to grayscale with only a single color space (equation d, Figure) where *res (x,y)* is the image in grayscale. After converting the image, the grayscale was replaced by a single color space, with black of lesser intensity and white of greater intensity [9].

A Gaussian filter was then used which gave the convolution, where, for each item on the input array, a Gaussian kernel was applied. The output is the filter operation based in a Gaussian function [14,19]. The kernel size was fixed to 7 x 7, so that the result was a smoother image. This operation was justified for a better bottom extraction [14], and with the reduction of some details and elements of the images, the edges of the red blood cells became more defined. Thresholding separated the grayscale in a given image such that the image was transformed into two grayscales groups: 0 (100% black) and 255 (100% white). For this process, it was necessary to set a cutoff or threshold to separate the two groups. The definition of this cutoff point was very important, because the images had different characteristics (eg, lighting and focus).

The Otsu thresholding technique [19] was chosen because it is widely used in the literature and the cutoff feature has an optimal, automatic threshold. The OTSU technique uses a nonparametric approach (not estimating the parameters of the model) and an unsupervised stop to find the automatic threshold in a given image [21]. The method contains the background and foreground pixel classes, represented by C1 and C2, respectively [19]. After the image was smoothed by the Gausssian filter, it was transformed into a binary image containing only foreground (blood cells) and background (rest of the image). With respect to malaria detection, the background is not important because most parasites lie within the cells of the prepared blood smears [5]. Morphological operations may be applied to images to enhance the geometric structures on a set of pixels. Erosion is a morphological technique that seeks to combine two sets by subtracting the vectors for the intersection sets of A and B (equation e, Figure 1).

The preprocessing transformation steps were necessary for locating the RBCs and restricting the search space of the algorithm. After the erosion, a subtraction was performed with the image converted to grayscale and the background was removed. The final result is shown in Figure 5.

### Training and Validation

The objective of the training and validation steps was to find a good classifier to recognize the parasite *Plasmodium falciparum* in the trophozoite (ring stage) in a given image. The training and validation of classifiers was performed according to the method by Viola and Jones [10] using an Intel Core i3-3217U (1.8 GHz 4096MB) computer.

For all the training sets, the following parameters were used: (1) the amount of memory was 1024, (2) a minimum hit rate of 0.99500, (3) a maximum false alarm rate of 0.400000, (4) basic, haar-like features mode [10,22], (5) a subwindow weight of 24, and (6) a subwindow height of 24.

Every image was classified as positive or negative, where positive referred to images infected with the *Plasmodium falciparum parasites* (trophozoite ring stage), whereas negative referred to white blood cells and empty RBCs (false positive). After classifying the images, the adaptive boosting algorithm was used to select the best features according to the stage of the cascade of classifiers [10]. The result of the training was a degenerative decisions tree with two positive and negative classes.

### Mobile App Development

Currently, the major developers of operating systems for mobile devices are Google Android, Apple IOS, and Microsoft Windows Phone [23-26]. Globally, Android is the most popular mobile operating system with about 1 billion users connected on various devices (tablets, mobile phones, televisions, etc) [24]. Android-based apps are mostly developed in Java [27]; however, apps can be also created in C/C++ using the Native Development Kit (NDK) [24].

We developed an automated, mobile device-based tool (Android) for the rapid and accurate diagnosis of malaria. The prototype development flow (pseudocode) is shown in Table 1.

**Table 1:** Prototype development flow.

Algorithm		
1: initialize camera		
2: set the camera resolution to 640 x 480 pixels		
3: **While** true **do**		
4:	view image in camera	
5:	perform to image scanning through windows 24 x 24 pixels (multi-scale)	
6:	**if** *find the object of interests* **then**	
7:		mark the object and count
8:	**end if**	
9:	**else** *keep looking*	
10: **end while**		
11: **if** *press the button return* **then**		
12:	close the application and release the camera	
13: **End if**		

Detection was performed by the mobile camera operating at a resolution of 640 x 480 pixels, with an average rate of 5 frames per second (FPS). Factors such as distance, lighting, and training set influenced the detection rate. While the camera is fixed, sight (focus) is adjusted manually on a light microscope. Parasites were detected on a frame-by-frame basis [10].

A second app was also developed that detects malaria in previously-acquired images. While the image analysis procedure is the same, the second app has a button that can upload an image rather than acquiring it with the device camera.

## Results

A set of experiments was designed to evaluate and quantify the accuracy of the developed tool in diagnosing malaria. The evaluation was binary, using positive (infected with *Plasmodium*-type parasites) and negative (not infected) classes. The acronyms used in the evaluation were positive sample (P), negative sample (N), parasites correctly detected as positive (TP), correctly identified as negative objects (TN), objects that are not parasites of the *Plasmodium sp* (FP), and positive parasites not detected by the classifier (FN).

In the field of artificial intelligence, metrics to evaluate performance are given. Here, the metrics of recall or sensitivity, specificity, precision rate (PR), and accuracy were used to evaluate the classifiers [28,29]. Recall or sensitivity measured the percentage of positive samples correctly classified as positive samples (equation f, Figure 1). Specificity measured the percentage of correctly identified negative samples out of the total number of negative samples (equation g, Figure 1). PR was the probability that a retrieved sample was relevant (equation h, Figure), whereas accuracy was the proximity degree between the value obtained and the true value (equation i, Figure 1).

Our system was tested using samples from the Microbiology Department (Parasitology Drassanes Unit) of Vall Hebron Hospital, Barcelona, Spain and the (microbiology lab) of Research Center Aggeu Magalhães (FIOCRUZ) Recife, Brazil. For these samples, the images were acquired using the mobile devices (Figure 4). The rest of the images were obtained from Wellcome Images [30,31].

As a preliminary experiment, the classifier was given 555 positive and 777 negative samples. A 10-fold cross-validation procedure was used for training and testing [10] using the adaptive boost algorithm. The results are shown in Tables 2 and 3.

**Table 2:** The 10-fold, cross-validation procedure on 10-, 15-, and 20-stage cascades.

Cascade	Iteration (K)	Specificity	Recall or sensitivity	Accuracy	PR^a^
10-stage					
	1	0.7075	0.7272	0.7096	0.2272
	2	0.9331	0.6428	0.9019	0.5373
	3	0.6414	0.8070	0.6596	0.2169
	4	0.8838	0.6545	0.8596	0.4000
	5	0.8731	0.7090	0.8557	0.3979
	6	0.8666	0.8727	0.8673	0.4363
	7	0.3383	0.6785	0.375	0.1101
	8	0.1626	0.8983	0.2461	0.1207
	9	0.6206	0.7857	0.6384	0.2000
	10	0.7960	0.8437	0.8019	0.3673
15-stage					
	1	0.9548	0.5090	0.9076	0.5714
	2	0.9892	0.4000	0.9269	0.8148
	3	0.9137	0.6964	0.8903	0.4936
	4	0.9634	0.7636	0.9423	0.7118
	5	0.9590	0.7142	0.9326	0.6779
	6	0.9482	0.9107	0.9442	0.6800
	7	0.8134	0.7627	0.8076	0.3435
	8	0.8599	0.6607	0.8384	0.3627
	9	0.8663	0.7500	0.8538	0.4038
	10	0.9234	0.7301	0.9000	0.5679
20-stage					
	1	0.9913	0.1636	0.9038	0.6923
	2	0.9956	0.1818	0.9096	0.8333
	3	0.9698	0.4464	0.9134	0.6410
	4	0.9655	0.7500	0.9423	0.7241
	5	0.9698	0.8035	0.9519	0.7627
	6	0.9590	0.8214	0.9442	0.7076
	7	0.9092	0.6491	0.8807	0.4683
	8	0.9652	0.4576	0.9076	0.6279
	9	0.9202	0.7500	0.9019	0.5316
	10	0.9650	0.4677	0.9057	0.6444

^a^PR: precision rate.

**Table 3:** Metric results reported as means (SDs).

Metric	Stage	Mean (SD)
Recall or sensitivity^a^		
	10	0.7619851 (0.092387416)
	15	0.6897741 (0.142576715)
	20	0.5491375 (0.244642849)
Specificity^b^		
	10	0.6823637 (0.2541448)
	15	0.9191804 (0.0558795)
	20	0.9611227 (0.0272376)
Average accuracy		
	10	0.6915384 (0.2224334)
	15	0.8938034 (0.0497291)
	20	0.9161538 (0.0225766)
PR^c^		
	10	0.3014110 (0.1450627)
	15	0.5627748 (0.1602032)
	20	0.6633560 (0.1068479)

^a^False positive rate.

^b^False positive rate.

^c^PR: precision rate.

## Discussion

### Principal Findings

**Figure 6:**
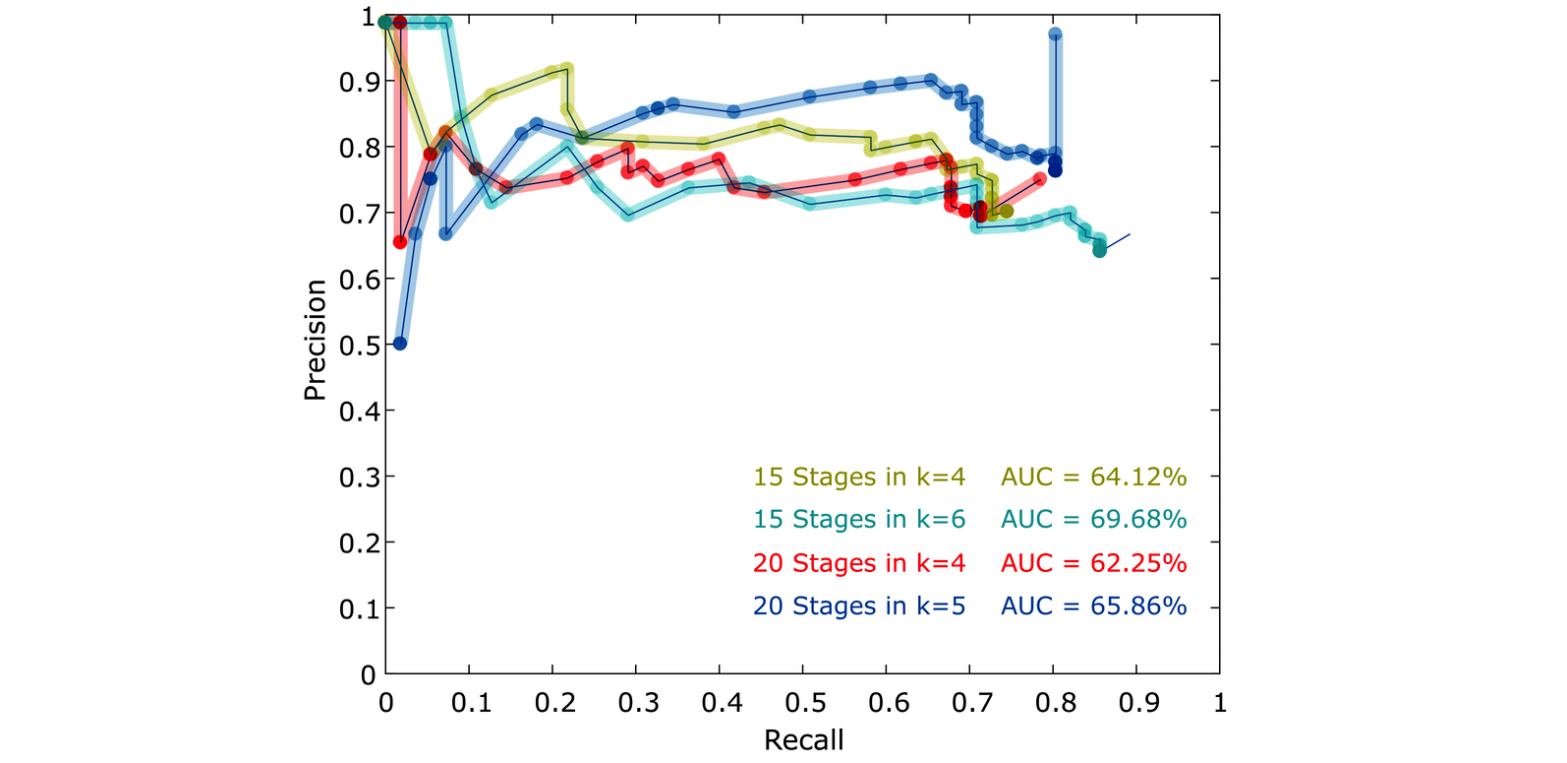
Precision-recall curve of the four iterations of the cross-validation method.

We developed a low-cost, automated, mobile device-based tool to diagnose malaria using image segmentation and artificial intelligence techniques. Based on the preliminary results, the classifier algorithm performed better using the 20-stage cascade with average specificity and accuracy values of 96% and 91%, respectively. Recall, however, was better using the 10-stage cascade, even though there were a high number of false positives that decreased the precision of the algorithm. Precision-recall curves were generated for the four best iterations (K) using the cross-validation method (Figure 6). The best area under the precision-recall curve was when recall and precision assumed a value of 1. After analyzing the curves, it was estimated that the 15-stage cascade in iteration 6 yielded the best result with respect to area under the curve (70%).

### Future Work

Currently, the highest level of accuracy was 91%. Based on these findings, the tool is feasible [12,13] and with new sets of positive images, higher rankings with respect to specificity and sensitivity are possible. In the future, variations and adaptations to the Viola and Jones [10] method may be enable detection of other species and stages of malaria development. In addition, new training algorithms such as neural networks [32], support vector machines [33], and even other image processing techniques [8,20] may be explored.

### Conclusions

The development of a low-cost, rapid, and accurate diagnosis tools for mobile phones and tablets that can be used in health centers in remote communities without the need for specific expertise could help break the accessibility barriers of low-resource countries. The tool that we developed can achieve this by virtue of its accessibility and on the spot, real-time diagnostic potential that may facilitate immediate treatment.

## Appendix

Multimedia Appendix 1 Screenshot of the Malaria System app beta version.

Multimedia Appendix 2 Video demo of the Malaria System.

## Abbreviations

FPR: false positives rate
PR: precision rate
RBCs: red blood cells
RCT: randomized controlled trial
RDT: rapid diagnostic tests
RGB: red, green, blue
ROC: receiver operating characteristic
TPR: true positives rate
