# Supplementary material for: The Malaria System MicroApp: A New, Mobile Device-Based Tool for Malaria Diagnosis
Source: JMIR Res Protoc. 2017 Apr 25;6(4):e70. doi: 10.2196/resprot.6758 (PMC5424126; doi:10.2196/resprot.6758)
Supplement: Multimedia Appendix 1 [file resprot_v6i4e70_app1.pdf]

Multimedia Appendix. Screenshot of the Malaria System app beta version.

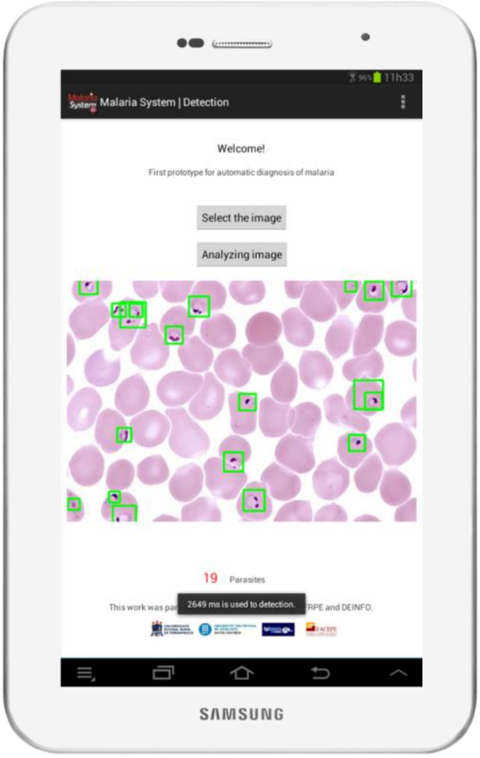

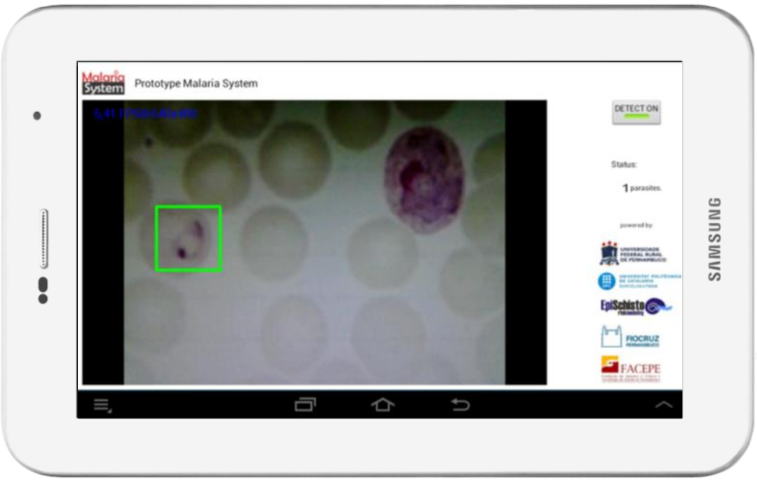

This is a Multimedia Appendix to a full manuscript published in the JMIR Research Protocols, for full copyright and citation information see http://dx.doi.org/10.2196/resprot.6758.
